# Supplementary material for: Evolutionary and Functional Analysis of Monoamine Oxidase F: A Novel Member of the Monoamine Oxidase Gene Family
Source: Genome Biol Evol. 2025 Jan 3;17(2):evae280. doi: 10.1093/gbe/evae280 (PMC11833248; doi:10.1093/gbe/evae280)
Supplement: evae280_Supplementary_Data [file evae280_supplementary_data.zip › Supplementary_Figure_S1.pdf]

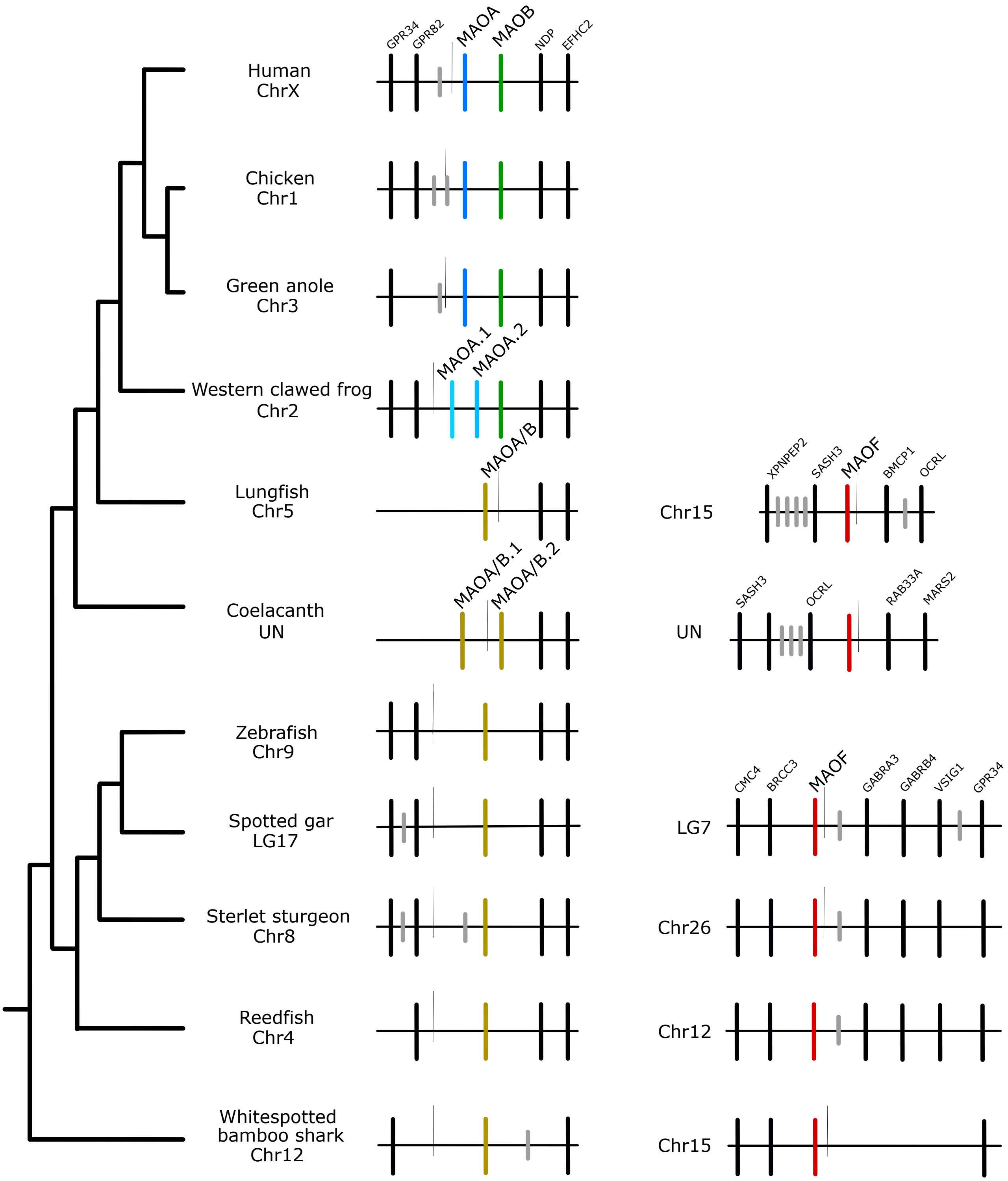

Supplementary Figure S1. Patterns of conserved synteny in the chromosomal regions harboring the monoamine oxidase genes of jawed vertebrates. Gray lines represent genes that do not contribute to conserved synteny. UN: Unplaced scaffold.
