# Supplementary material for: Evolutionary and Functional Analysis of Monoamine Oxidase F: A Novel Member of the Monoamine Oxidase Gene Family
Source: Genome Biol Evol. 2025 Jan 3;17(2):evae280. doi: 10.1093/gbe/evae280 (PMC11833248; doi:10.1093/gbe/evae280)
Supplement: evae280_Supplementary_Data [file evae280_supplementary_data.zip › Supplementary_Figure_S2.pdf]

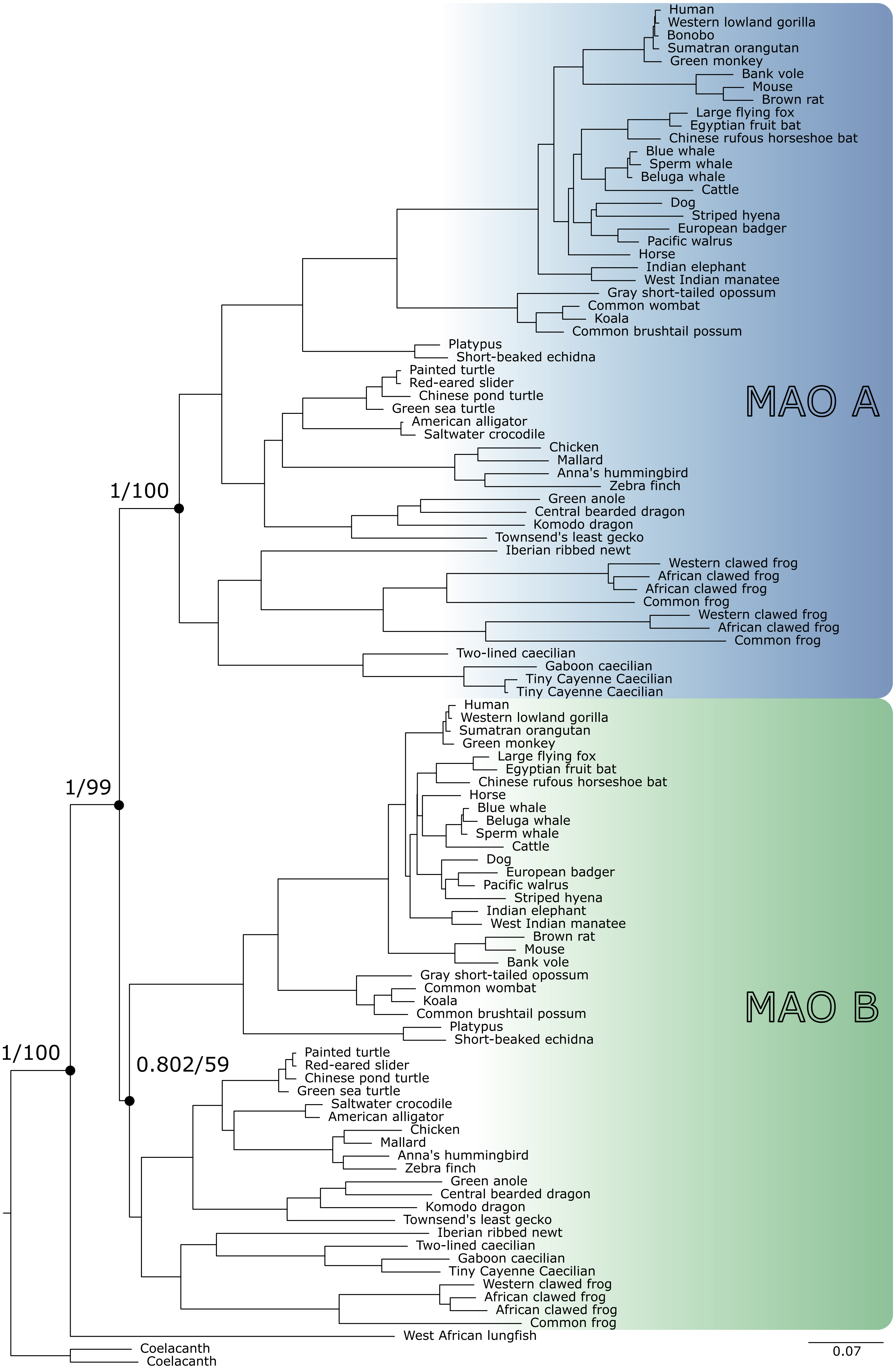

Supplementary Figure S2. Maximum-likelihood tree showing relationships among monoamine oxidases of tetrapods. Numbers on the nodes correspond to support from the abayes and ultrafast bootstrap values. The scale denotes substitutions per site, and shading represents gene lineages. MAO A/B nucleotide sequences from the West African lungfish (*Protopterus annectens*) and coelacanth (*Latimeria chalumnae*) were used as outgroups.
