# Supplementary material for: Evolutionary and Functional Analysis of Monoamine Oxidase F: A Novel Member of the Monoamine Oxidase Gene Family
Source: Genome Biol Evol. 2025 Jan 3;17(2):evae280. doi: 10.1093/gbe/evae280 (PMC11833248; doi:10.1093/gbe/evae280)
Supplement: evae280_Supplementary_Data [file evae280_supplementary_data.zip › Supplementary_Figure_S3.pdf]

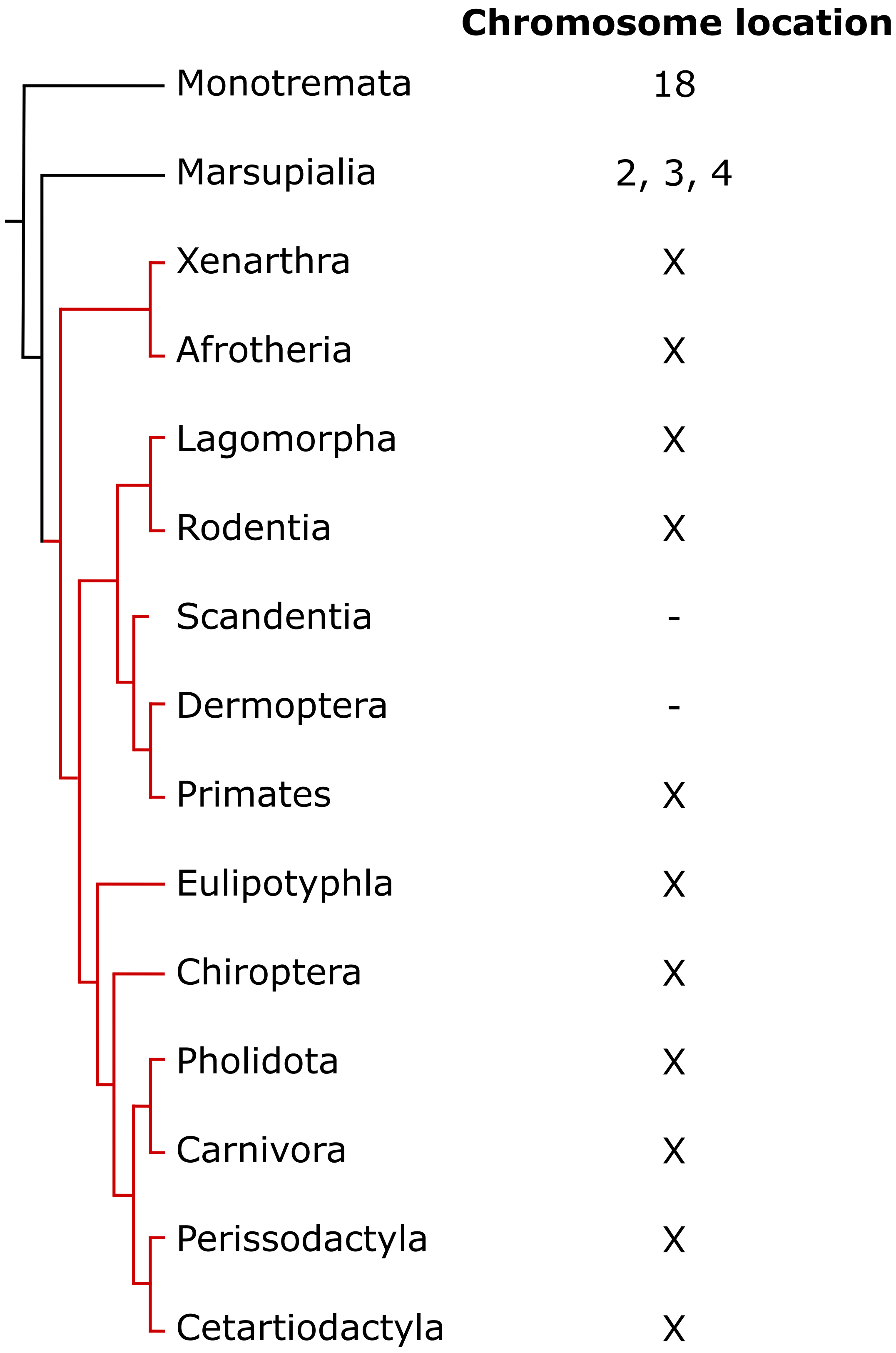

Supplementary Figure S3. Chromosomal location of MAO A and MAO B genes in mammals. As is shown, the translocation of MAO genes from an autosome to chromosome X occurred in the last common ancestor of placental mammals between 160 and 99 million years ago (Kumar et al. 2022). The tree topology was obtained from the literature (Esselstyn et al. 2017). Placental mammals are indicated with red branches. - represents lack of information regarding chromosomal location
