# Supplementary material for: Evolutionary and Functional Analysis of Monoamine Oxidase F: A Novel Member of the Monoamine Oxidase Gene Family
Source: Genome Biol Evol. 2025 Jan 3;17(2):evae280. doi: 10.1093/gbe/evae280 (PMC11833248; doi:10.1093/gbe/evae280)
Supplement: evae280_Supplementary_Data [file evae280_supplementary_data.zip › Supplementary_Figure_S4.pdf]

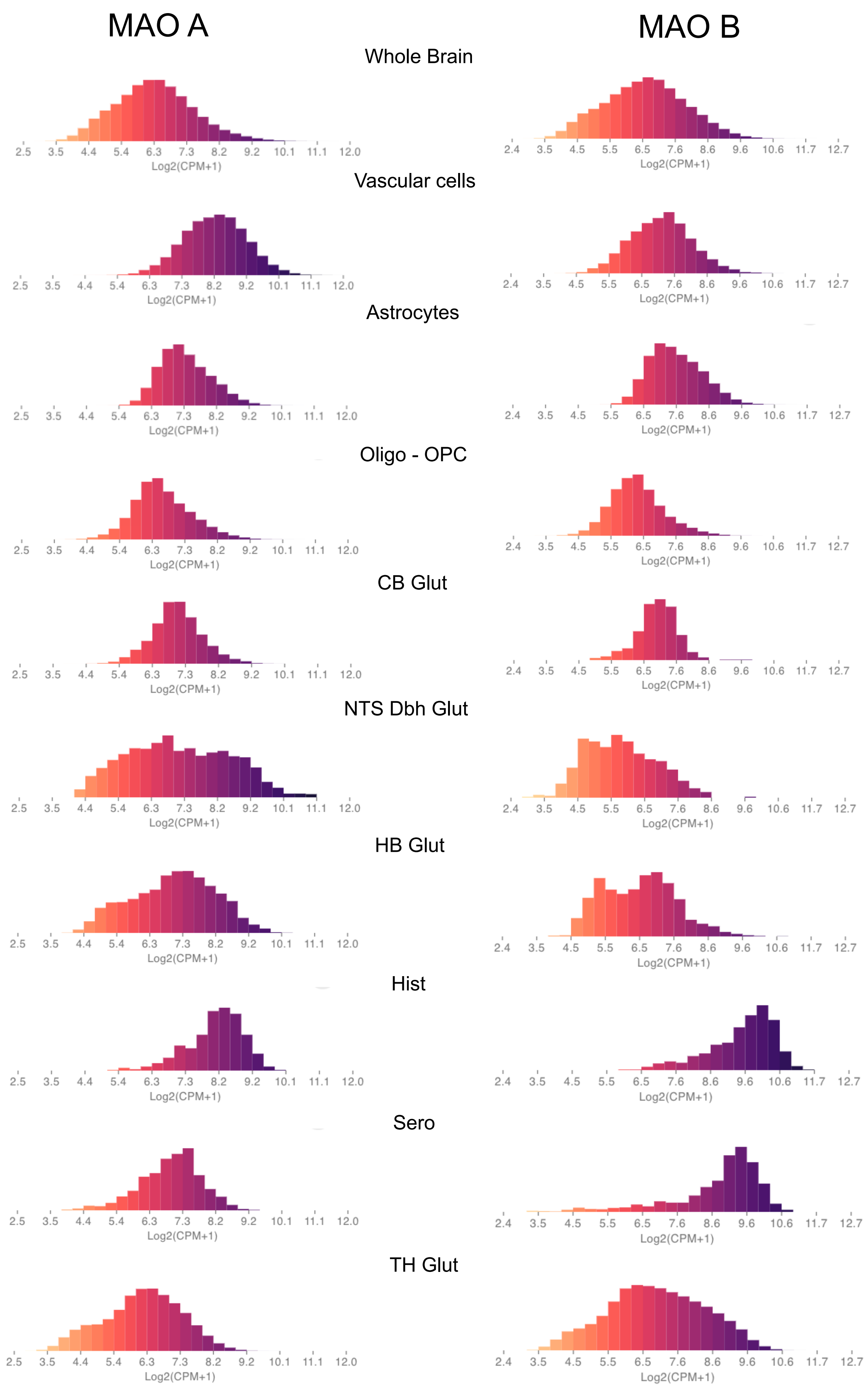

Supplementary Figure S4. Expression of MAO A and MAO B in the mouse brain.
Distribution of expression values of MAO A and MAO B transcripts in different mouse brain cell types. Oligo: oligodendrocytes, OPC: Oligodendrocyte precursor cells, CB: Cerebellum, Glut: Glutamatergic, NTS: Nucleus of the solitary tract, Dbh: Dopamine-β-hydroxylase, HB: Habenula, Hist: Histaminergic, Sero: Serotonergic, TH: Thalamus. These graphs were obtained from The Allen Brain Cell Atlas (https://portal.brain-map.org/atlases-and-data/bkp/abc-atlas).
